# The Prevalence of Potential Drug-Drug Interactions in CKD-A Retrospective Observational Study of Cerrahpasa Nephrology Unit

**DOI:** 10.3390/medicina58020183

**Published:** 2022-01-26

**Authors:** Andleeb Shahzadi, Ikbal Sonmez, Cagla Kose, Burhaneddin Oktan, Selma Alagoz, Haktan Sonmez, Adil Hussain, Ahmet Gokhan Akkan

**Affiliations:** 1 Department of Medical Pharmacology, Cerrahpasa Medical Faculty, Istanbul University-Cerrahpasa, Istanbul 34098, Turkey; shahzadiandleeb@yahoo.com (A.S.); mikbalsonm@gmail.com (I.S.); cagla.k.kose@gmail.com (C.K.); oktanburhaneddin@gmail.com (B.O.); haktansonmezz@gmail.com (H.S.); 2 Department of Medical Pharmacology, Medical Faculty, Halic University, Istanbul 34098, Turkey; 3 Department of Internal Medicine, Division of Nephrology, Cerrahpasa Medical Faculty, Istanbul University-Cerrahpasa, Istanbul 34098, Turkey; 4 Department of Nephrology, Istanbul Training and Research Hospital, Health Sciences University, Istanbul 34098, Turkey; 5 Liver Centre, District Headquarters Hospital-Faisalabad, Faisalabad 38800, Pakistan; adilhussain9600@gmail.com; 6 Department of Medical Pharmacology, Medical Faculty, Bezmialem Vakif University Hospital, Istanbul 34098, Turkey; gakkan@bezmialem.edu.tr

**Keywords:** chronic kidney disease, drug-drug interactions, polypharmacy, hypertension, hypotension

## Abstract

*Background and Objectives:* Chronic kidney disease (CKD) is usually linked with polypharmacy and patients are invariably at risk of complex medication regimens. The present study was designed to estimate the potential drug-drug interactions (pDDIs) through the prescription patterns provided to patients of the Nephrology Transplant Unit of Cerrahpasa Medical Faculty patients. *Materials and Methods:* 96 patients were included in the study. pDDIs among every combination of the prescribed drug were analyzed using the Thomson Reuters Micromedex. *Results:* We found 149 pDDIs making 2.16 interactions per prescription with incidence rates of 69.7%. Approximately 4.1% of interactions were of significant severity, 75.1% moderate severity, and 20.8% were classified as minor pDDIs. The most frequent interactions were found between iron and aluminum, calcium or magnesium-containing products (21.37%), calcium channel blockers and beta-blockers (8.96%); and aspirin and aluminum, calcium, or magnesium-containing products (7.58%). We identified 42 drug pairs with probability of clinical significance. The most commonly reported clinical outcomes of the pDDIs were hypo- or hypertension (39.24%), decreased drug efficacy (24.05%), and arrhythmia (9.49%). Aluminum, calcium, or magnesium-containing drug products (33.10%) constituted the primary class of drugs involved in interactions. *Conclusions:* This study showed pharmacodynamics (49%), pharmacokinetics (42.94%) interactions, polypharmacy and gender as determinant of pDDIs. A comprehensive multicenter research is required to decrease the morbidity and ease the state burden.

## 1. Introduction

Chronic kidney disease (CKD) is a challenging issue for medical science. Renal impairment is often left underdiagnosed; however, efforts are being made to improve the detection and management of patients suffering from this disorder [1]. The Turkish Society of Nephrology 2008 annual registry data indicate that the incidence of end-stage renal disease (ESRD) in Turkey has increased up to four-fold since 2000 [2]. The prevalence of CKD in Turkey is found to be 15.7% [3]. The National Health and Nutrition Examination Survey (NHANES) showed that 38% of the elderly (>65 years) population suffer from CKD [4]. The literature shows that morbidities and mortalities are associated with CKD as this population has an increased risk of diabetes and cardiovascular events [5,6]. These factors are the primary cause of end-stage renal disease and play an essential role in chronic kidney disease (CKD) prognosis. The increase in hypertension and diabetes leads to an increased frequency of occurrence CKD [6,7].

Despite the presence of numerous guidelines regarding drug dosing for patients with reduced kidney function, there is inadequate evidence to make decisions on many commonly used drugs. 

Patients with ESRD are at high risk of developing drug-related problems due to polypharmacy, multiple co-morbidities, and frequent medication changes [8]. Polypharmacy has a significant role in drug interaction, being one of the leading causes of healthcare morbidity and mortality. It is estimated that the incidence of clinical drug interaction ranges from 3 to 5% in patients taking few medications whereas increases to 20% in patients receiving 10–20 medications. ESRD patients receiving hemodialysis are on complex drug regimens and almost 10–12 medications per day [9].

Polypharmacy is described as the concomitant prescription of five or more drugs [10] and is a considered as 100% risk of pDDIs with eight or more drugs. The elderly population is at risk of polypharmacy with an increasing chance of adverse drug reactions (ADRs) two and five medications [11,12]. Drug-drug interactions can result in life-threatening and even lethal toxicities. Almost 10% of admissions in general hospitals are due to potentially severe drug-drug interactions caused by improper use and combinations of drugs [13]. Furthermore, CKD patients usually comprise the elderly population that has diabetes, hypertension, and other medical complications that pose a high risk for potentially severe drug interaction [14].

Previous studies assessed the prevalence and severity of pDDIs by using different drug-drug interaction programs among CKD patients from Brazil [15], India [9], Pakistan [16], Palestine [17], Nigeria [18,19] and Spain [14]; however, there are no published studies evaluating the prevalence of pDDIs among CKD patients of Turkey.

Medication-related problems are prevalent in patients with chronic kidney disease. An interdisciplinary approach requires the identification, prevention, and management of these quandaries. Based on the above information, the following article focuses on pDDIs faced in nephrology, help, prevent and reduce the morbidity associated with this class of patients.

## 2. Materials and Methods

### 2.1. Design-Setting

A retrospective, observational study was conducted on 96 end-stage renal transplant patients on the cadaveric renal transplant waiting list with GFR < 15. This study was conducted in collaboration with the Medical Pharmacology Department and Nephrology Unit Cerrahpasa Medical Faculty.

**Figure 1:**
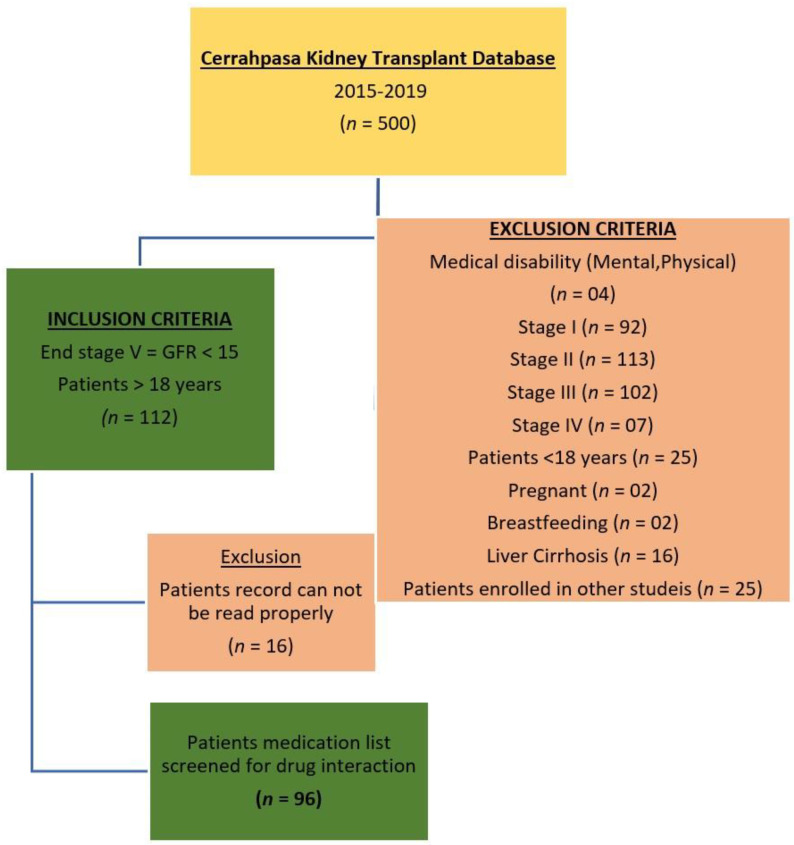
Flow diagram indicating the patient exclusion criteria.

In the duration of six months (December 2009 to May 2010) we reviewed nephrology transplant unit Cerrahpasa patient records from 2015–2019. The patients included in this study were males and females more than 18 years old and on more than two medications. Patients with medical disabilities (mental or physical impairment) were excluded. Prescriptions issued for hospitalized patients along with medication information were collected by interviewing the patients and medical professionals, including length of stay in the hospital and patient demographics (age and sex). The usage of the medications of each individual patient was cross-checked with the nurse’s administration record (Figure 1).

### 2.2. Data Collection

The drug combination in every prescription was analyzed using the Thomson Reuters Micromedex® DrugReax® system and www.drugs.com for potential DDIs among the prescription medications. This computer program details all the possible interactions and declares whether the information is available on a particular drug within a group of medications. It also indicates the clinical significance of the interaction, whether the interaction has been fully established in the literature, and also provides citations. The number of drug pairs was determined according to the number of drugs per prescription. DDIs were categorized according to pharmacokinetics, pharmacodynamics parameters, and severity (minor, moderate, and significant). The potential DDIs have been evaluated with clinical risk factors.

### 2.3. Classification of Potential Drug-Drug Interaction

Based on prescription characterization, the DDIs were identified and classified using www.drugs.com (Drug-Reax database) and Thomson Reuters Micromedex. DDIs were classified according to severity as (1) major–the effects are potentially life-threatening or capable of causing permanent damage; (2) moderate–the effects may cause a decline in patient’s clinical state and supplementary treatment or prolongation of hospital stay; (3) minor–the effects are usually mild. 

### 2.4. Evaluation of DDI Frequency 

The frequency of DDIs was estimated by using the following formula [20]:

Frequency of DDIs = Total no. of DDIs/Total no. of patient × 100

### 2.5. Statistical Analysis

The descriptive and inferential statistical analysis was performed by using IBM SPSS software (version 26). The correlation between pDDI and polypharmacy was analyzed with Pearson correlation test and then Logistic regression was applied to identify the association between the pDDIs and the patient characteristics. In the model, the exposure to pDDIs was considered dependent variable (0 = absent, 1 = present). Further variables used in the model as predictors of pDDIs were as follows: gender (1 = female, 2 = male), age (0 ≤ 60 years, 1 ≥ 60 years), number of prescribed drugs (0 ≤ 5, 1 ≥ 5), diabetes (1 = Yes, 0 = No), other disease (1 = yes 0 = No) and hypertension (1 = Yes, 0 = No). Kruskal Wallis test was used to describe the association between the pDDI-occurrence and prescribed medicines. Moreover, the frequency of the incidence of various drug interactions was statistically compared by using Mann Whitney U test. The data was considered statistically significant at *p* < 0.05.

## 3. Results

A total of 96 patient case records were reviewed on the cadaveric renal transplant waiting list during a six-month study period in which 41 patients (42.7%) were male; and 55 (57.3%) were female. The average age of the patients was 53 and ranged from 26 to 77 years. The majority of the patients enrolled had past medical problems such as chronic kidney disease (80.2%), hypertension, and diabetes mellitus (25%). The demographic characteristics of patients with pDDIs are presented in Table 1.

**Figure 2:**
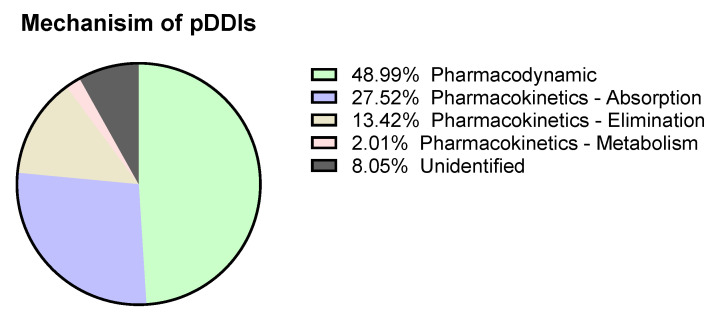
Mechanism of pDDIs.

Out of 96 reviewed patient’ case records, 67 (69.7%) patients showed potential pDDIs (*p* < 0.001). 149 pDDIs were identified, out of which 31 were minor, 112 were moderate, and six were significant or severe (*p* < 0.001; Table 2). We found 49% pharmacodynamics interactions and 42.9% of pharmacokinetics interactions (Figure 2). 

The average number of medicines and indications per patient was 6.25 and 2.3, respectively. The statistical significance of the association of pDDIs with the prescribed medicines and the occurrence of various drug interactions is presented in Table 3.

The most frequent interactions were found between iron and aluminum, calcium, or magnesium-containing products (21.37%); calcium channel blockers and beta-blockers (8.96%), and aspirin and aluminum, calcium, or magnesium containing products (7.58%). The most commonly reported clinical outcomes of the pDDIs were hypo or hypertension (39.24%), decreased drug efficacy (24.05%), and arrhythmia (9.49%). Aluminum, calcium, or magnesium-containing drug products (33.10%) constituted the primary class of drugs involved in interactions. 

Pearson correlation coefficient of polypharmacy and PDDI was found to be 0.34 (0.14–0.49 with 95% confidence interval, *t* = 3.45 *p* = 0.0008). Logistic regression showed two determinants for pDDI in end stage CKD patients were significant (use of more than five drug and age) other characters were not significant (*p* > 0.05). The odds of pDDIs interactions in patients with more than five drugs were higher by factor of 6.52 than odds of patients with less than five drugs (*p* < 0.001). The odds of pDDIs in men were higher by factor of 2.09 than odds of potential drug interactions in women (*p* = 0.15). 

Patients with three different indications found to have the highest number of drug prescribed (6.9) and the maximum pDDIs (3.0) was found in one patient suffering with five different diseases (Table 4). In addition to CKD, 80.2% of patients had hypertension, 25% diabetes mellitus, 17.8% hypertension + diabetes mellitus, and 12.5% of other diseases (Table 4).

The possible clinical symptoms associated with drug-drug interaction in CKD patients were listed in Table 5. The most commonly observed adverse effect was decreased drug efficacy by 25%; followed by hypertension Table 5).

## 4. Discussion

The scientific literature on polypharmacy associated with chronic kidney disease (CKD) patients is still limited. This population is continually at risk of complex medication regimens and compromised adherence with significant increase in healthcare costs, length and frequency of hospitalization, with a unfavorable impact on quality of life [21]. A comprehensive interdisciplinary approach is required to identify, prevent, and manage problems associated with polypharmacy [22]. 

The following study highlights the detection of pDDI prevalence in 96 end-stage renal transplant patients (41 male and 55 female) of the Nephrology Department of Cerrahpasa Medical Faculty Hospital, Istanbul University; by using computer programs like www.drugs.com and Thomson Reuters Micromedex.

In the current study, we analyzed the prescriptions of 96 CKD patients, which showed multiple drug regimens. One of these prescriptions had 12 medicines, and the number of drugs prescribed to the study population have an average of 6.25 medications.

A recent study conducted in Spain on CKD with 957 prescribed medications and 928 pDDIs [14]. In the present study, pDDIs interactions were higher in patients with more than five drugs. It has been estimated that the prevalence of DDIs in CKD patients ranged between 56.9% and 89.1% [23]. Another study conducted in Jordan showed that 96% of polypharmacy patients at outpatient clinics have at least one pDDI. Almost half of the detected interactions involved cardiovascular medications [24]. We also found out of 149 pDDIs 77 were comprised of cardiovascular events in CKD patients.

We found 69.7% pDDIs in the medications of CKD patients with 4.1% significant severity. However, most drug interactions were moderate (75.1%) to mild (20.8%). Severe drug-drug interactions are life-threatening and require medical treatment or intervention to minimize or prevent severe adverse effects. Moderate drug-drug interactions may lead to worsening of the condition or might require a change in the therapy. Mild drug-drug interactions restrict the clinical outcomes. The manifestations constitute an increase in adverse effects, but these usually do not require a change in the therapy. 

Similarly, Doubova Dubovaet et al. [25] conducted a study on the prescriptions of 624 ambulatory patients of a family clinic in Mexico City, and found that 80% of the patients had one or more pDDIs and 3.8% of patients’ prescriptions had a drug combination that should have been avoided. A cohort study by van Dijk, et al. [26] on 2,335 patients of Dutch nursing home residents found that 32% of the patient’s drug combinations had clinically adverse outcomes. 

In our study, the average number of medications per prescription was higher than that conducted on ambulatory patients of family medicine clinics in Mexico, which was 5.9 per patient [26]. A study conducted by Glintborg, et al. [27] on 200 patients showed the median number of drugs was eight, ranging from 1 to 24. Similarly, another study conducted by Rama et al. on the patients of a nephrology ward of a south Indian tertiary care hospital showed the median number of medications per patient was 11, indicating polypharmacy as a risk factor for pDDI and a predictable risk factor for medication errors [9]. Polypharmacy reinforces the problems relating to pDDI in renal failure patients, which needs continuous efforts to optimize drug therapy.

We also found that polypharmacy in CKD patients was due to the prevalence of other diseases; for example, 80.2% of the patient population of this study had hypertension, 25.0% of patients were suffering from diabetes mellitus; and 17.7% had both hypertension and diabetes mellitus, while the remaining 12.5% with other complications.

Combination disease patients (hypertension and diabetes mellitus) showed the highest number of pDDIs, followed by the patients suffering from diabetes mellitus alone. This interaction can be subjected to the dramatic increase in the number of medicines over the past few years to manage hypertension and diabetes. The risks for possible drug interactions in diabetic patients can be hyperglycemia, hypoglycemia, or other deleterious effects, which exponentially increase with the number of medications [28]. Many of these interactions can be avoided by monitoring and adjusting the serum dosage, glycemic control, through clinical or laboratory control, and or avoiding that particular combination. 

The most common interactions seen in our study were among electrolytes and iron. The patients with solitary hypertension and combined diabetes had the most significant number of moderate interactions, followed by diabetes alone (minor DDIs). Moreover, the literature indicated that CKD patients with insulin resistance or hyperinsulinemia are more at risk of developing cardiovascular events [29]. The large number of drugs prescribed to patients with hypertension and diabetes mellitus can be attributed to beta-blockers, ACE inhibitors, hypolipidemic agents, calcium channel blockers, insulin, and anticoagulants to decrease the risk of various complications.

Interaction during the concomitant administration of drugs may not manifest itself immediately but can be the basis of a decrease or increase in the efficacy of drugs, leading to therapeutic failures and potential adverse events [30,31]. In the current study, most of the drug-drug interactions were of a pharmacodynamics type (50.34%), followed by pharmacokinetic interactions at absorption, elimination, and metabolism. Furthermore, Alomar [30], reported an interaction between theophylline and tacrolimus, an immunosuppressive drug used for prophylaxis and allograft rejection treatment. The study showed an increase in the tacrolimus area under the curve after administering low-dose theophylline by inhibiting CYP3A4. 

In this study we found 37 drugs on the prescription of 96 patients which showed the 42 drug pairs identified with a probability of clinical significance is shown in Table 3. The occurrence of statistically significant interactions makes it inferential for any population. Some drugs were on one or two prescription like pentoxifylline, perindopril and prednisolone, the incidence of pDDI was not statistically significant, and we consider it due to small sample size (Table 3). For proper management of CKD patients and improving optimal health a state study is essential to determine differences across hospitals and regions. 

## 5. Conclusions

The present study recorded a high prevalence of pDDIs in nephrology wards and identified 149 pDDIs, of which the most prevalent were of moderate to significant severity. We found a statistically significant correlation between pDDI and polypharmacy in patients with end-stage chronic kidney disease. In these patients the clearance of drug is decreased. Hence, both direct pharmacokinetic parameters and indirect pharmacodynamic parameters of drugs alter. This creates a suitable environment for drug-drug interactions. This study also indicated that the number of prescribed medications ≥5 and poorly gender as determinant of pDDIs.

The result of the present study serves as a warning for the interdisciplinary team. The chance of pDDIs can be subsided by choosing alternative medication, dose adjustment, and patient monitoring. For all of the above, the easiest way to decrease the frequency of pDDIs is to decrease the number of prescribed medicines. However, it is sometimes difficult to reduce the number of drugs prescribed for patients with CKD; consequently, it is crucial to thoroughly observe the patients for adverse events. The assistance of clinical pharmacists is of great importance in this process to provide information for making better decisions, ensure the practical, rational, and safe use of medicines, improve the quality of treatment, and reduce the risks for CKD patients.

## 6. Limitations 

The potential limitations of this study is the lack of control group we only included end stage renal transplant patients. Additionally, it is a single centered study due to lack of funding therefore, these study findings should be carefully generalized.

## Figures and Tables

**Table 1:** Demographic characteristics.

Number of Patients	96
Male, *n* (%)	41 (42.7)
Female, *n* (%)	55 (57.3)
Average age (years)	53 (26–77)
Diagnosis	
Hypertension, *n* (%)	77 (80.2)
Diabetes Mellitus, *n* (%)	24 (25.0)
Hypertension + Diabetes Mellitus, *n* (%)	17 (17.7)
Other Diseases	12 (12.5)
Number of indication	Patients, *n* (%)
1	10 (10.4)
2	56 (58.3)
3	22 (22.9)
4	7 (7.3)
5	1 (1.0)
Average indication	2.3

**Table 2:** The number of patients with minor, moderate, and major drug interactions.

Interaction	Number of Patients
DDIs	67 (69.8%)
No DDIs	29 (30.2%)
Degree	Number of DDIs
Minor	31 (20.8%)
Moderate	112 (75.2%)
Major	6 (4.0%)
Total	149

**Table 3:** Statistical analysis of the incidence of pDDIs. Asterisks are placed on the p values that are statistically significant.

Medicine Prescribed	*p* Value (Kruskal Wallis Test)	Interactions	*p* Value (Mann Whitney U Test)
Electrolytes	0.000 *	Iron + Electrolytes	0.000 *
Iron	0.000 *	Calcium channel blockers + β-blockers	0.000 *
β-Blockers	0.000 *	Aspirin + Electrolytes	0.000 *
Non-steroidal anti-inflammatory drugs	0.000 *	β-blockers + NSAIDs	0.000 *
Calcium Channel blockers	0.000 *	Calcium channel blockers + NSAIDs	0.000 *
Aspirin	0.000 *	Ascorbic acid + Cyanocobalamin	0.000 *
Calcium	0.000 *	α-blockers + β-blockers	0.001 *
Lansoprazole	0.000 *	Antidiabetics + β-blockers	0.001 *
Antidiabetics	0.010 *	Iron + Lansoprazole	0.001 *
Ascorbic acid	0.010 *	Lansoprazole + Antacids	0.001 *
Cyanocobalamin	0.010 *	Angiotensin receptor blockers + NSAIDs	0.003 *
Angiotensin Receptor Blockers	0.022 *	Verapamil + Calcium	0.003 *
Antacids	0.022 *	Aspirin + Insulin	0.011 *
Angiotensin converting enzyme inhibitors	0.107	Calcium + Hydrochlorothiazide	0.011 *
Sucralfate	0.022 *	Doxazosin + Nifedipine	0.011 *
α-Blockers	0.022 *	Sucralfate + Electrolytes	0.011 *
Verapamil	0.022 *	ACE inhibitors + Furosemide	0.040 *
Furosemide	0.049 *	Furosemide + Sucralfate	0.040 *
Thiazide	0.482	Methyldopa + Iron	0.040 *
Doxazosin	0.107	NSAIDs + Thiazide	0.040 *
Hydrochlorothiazide	0.107	ACE inhibitors + Angiotensin receptor blockers	0.148
Insulin	0.107	ACE inhibitors + Thiazide	0.040 *
Nifedipine	0.107	Amlodipine + Diltiazem	0.148
Amlodipine	0.228	Amlodipine + Simvastatin	0.148
Diltiazem	0.228	Antidiabetics + Thyroxine	0.148
Methyldopa	0.228	Aspirin + Calcium	0.148
Atorvastatin	0.482	Aspirin + Diclofenac	0.148
Clopidogrel	0.482	Aspirin + Perindopril	0.148
Diclofenac	0.482	Aspirin + Prednisolone	0.148
Indapamide	0.482	Aspirin + Ramipril	0.148
Levothyroxine	0.482	Atorvastatin + Verapamil	0.148
Perindopril	0.482	Calcium acetate + Calcium aspartate	0.148
Prednisolone	0.482	Calcium + Indapamide	0.148
Ramipril	0.482	Calcium + Levothyroxine	0.148
Simvastatin	0.482	Calcium + NSAIDs	0.148
Thyroxine	0.482	Clopidogrel + NSAIDs	0.148
Pentoxifylline	0.482	Diltiazem + β-blockers	0.148
		Pentoxifylline + NSAIDs	0.148

* indicates statistical significance (*p* < 0.05).

**Table 4:** Showing average number of medicines and drug interaction in relation to disease.

Diagnosis	Number of Patients	Avg. Number of Drug	Avg. Interaction Number
Hypertension	77 (80.2%)	6.4	1.6
Diabetes mellitus	24 (25.0%)	7.0	2.6
Hypertension + diabetes Mellitus	17 (17.7%)	7.1	2.7
Other diseases	12 (12.5%)	5.3	0.8

**Table 5:** Classification of possible clinical effects resulting from pDDIs.

pDDIs	Number	Percent (%)
Decreased drug dffects	38	25.45
Hypertension	35	23.45
Hypotension	27	18.05
Arrhythmia	15	10.05
Bleeding risk	11	7.50
Hypoglycemia	8	5.50
Others	15	10.00
Total	149	100.00

## Data Availability

The authors confirm that the data supporting the finding of current study are available within the article. The raw data of this study are available from the corresponding author, upon reasonable request.
